# Mortality of Surgical Operations in the Upper Lake States, Compared with That of Other Regions

**Published:** 1876-10

**Authors:** Edmund Andrews

**Affiliations:** Professor of Princples and Practice of Surgery in Chicago Medical College


### THE MORTALITY OF SURGICAL OPERATIONS IN THE UPPER LAKE STATES, COMPARED WITH THAT OF OTHER REGIONS.

By EDMUND ANDREWS, A.M., M.D.,

Professor of Principles and Practice of Surgery in Chicago Medical College,

Assisted by Thos. B. Lacey, M.D.,

Assistant Surgeon in the National Soldiers' Home.

(Continued.)

### AMPUTATION AT THE KNEE JOINT.

This operation has evidently not been a favorite in the Lake States, as I have no cases of it reported to me. Abroad the following statistics are found:

PRIMARY.

| AUTHORITIES. | CASES. | DIED. |
|---|---|---|
| Boston City Hosp. Rep. Dr. Cheever | 1 | 0 |
| St. Bartholomew's Hosp., 1863–71 | 2 | 0 |
| Mass. Gen. Hosp | 5 | 3 |
| Circular No. 6, Surg. Gen. U. S. A. | 49 | 16 |
| Crimean War, Schmidt's Jahrbücher, Vol. 156, p. 250 | 39 | 31 |
| German-French War, " " " " " " | 1 | 1 |
| American Jour. Med. Sci., 1868, pp. 333, 555. Dr. Brinton | 109 | 47 |
| Totals | 206 | 98 |

Mortality, 48 per cent.

INTERMEDIARY AND SECONDARY COMBINED.

| AUTHORITIES. | CASES. | DIED. |
|---|---|---|
| Dr. D. W. Cheever, Boston | 1 | 1 |
| St. Bartholomew's Hosp., 1863–71 | 1 | 0 |
| Siege of Strassburg, 1870–71. Dr. Herrgolt | 3 | 3 |
| Crimean War, Schmidt's Jahrbücher, Vol. 156, p. 250 | 8 | 7 |
| German-French War, " " " " " " | 5 | 4 |
| American Jour. Med. Sci., 1868, pp. 333, 555. Dr. Brinton | 68 | 27 |
| Totals | 86 | 42 |

Mortality, 49 per cent.

PATHOLOGICAL.

| AUTHORITIES. | CASES. | DIED. |
|---|---|---|
| Guy's Hospital Reports | 1 | 0 |
| St. Bartholomew's Hosp., 1863–71 | 6 | 2 |
| Mass. Gen. Hosp. | 11 | 4 |
| American Jour. Med. Sci., 1868, pp. 333, 555. Dr. Brinton. | 92 | 19 |
| British Army Med. Rep. | 1 | 0 |
| Prof. Billroth, Arch. klin. Chir., B. x. | 7 | 6 |
| Lücke, Deutsch. Zeitschrift für Chir., Bd. ii. | 1 | 0 |
| Totals | 119 | 31 |

Mortality, 26 per cent.

TIME OR CAUSES IMPERFECTLY STATED.

| AUTHORITIES. | CASES. | DIED. |
|---|---|---|
| St. George's Hospital, London | 7 | 3 |
| Circular No. 3, Surg. Gen. U. S. Army, Traumatic | 3 | 0 |
| " " 6, " " " " " | 73 | 51 |
| Bericht k. k. allg. Krankenhaus, Wien | 3 | 2 |
| Statist. des Hôp. de Paris, 1861–2–3 | 2 | 1 |
| Lücke. Deut. Zeitschrift, B. 2 | 2 | 1 |
| Zurich Hosp., 1860–67 | 1 | 1 |
| Arch. klin. Chir., Bd 17, S. 510 | 38 | 11 |
| Totals | 129 | 70 |

Mortality, 54 per cent.

SUMMARY OF AMPUTATIONS AT THE KNEE JOINT ABROAD.

| | CASES. | DIED. | PER CT. MORT. |
|---|---|---|---|
| Primary | 206 | 98 | 48 |
| Intermediary and Secondary combined | 86 | 42 | 49 |
| Pathological | 119 | 31 | 26 |
| Conditions imperfectly stated, but cases nearly all military | 129 | 70 | 54 |

The pathological cases here are nearly all those called by Brinton "secondary pathological;" that is, cases which are more or less chronic. The importance of the distinction is that amputations in the acute inflammatory stage of the knee joint are very fatal, whether performed at the knee or just above it, and should be avoided if possible.

OPINIONS OF AUTHORS.

Amputation at the knee joint, instead of at the lower third of the thigh, was first performed by Fabricius Hildanus, in

1581, and re-introduced to the profession mainly by the efforts of Velpeau, Markoe and Brinton. It was first performed in America by Prof. Nathan Smith.

The operation was at first mainly performed by leaving the condyles in the stump. Mr. Carden, of Great Britain, introduced as an improvement the plan of sawing off the articular portion of the condyles, and the statistics of that country apparently showed about six per cent. more safety by that method, but Dr. Brinton, (Am. Jour. Med. Sci., 1868,) adds the American statistics, which change the result, and render the two methods almost exactly equal, as may be seen in the following figures:

| CONDYLES LEFT. | CASES. | DIED. | PR.CT.MORT. |
|---|---|---|---|
| American Cases | 45 | 12 | 27 |
| Foreign Cases | 34 | 10 | 29 |
| Totals | 79 | 22 | 28 |

| CONDYLES REMOVED. | CASES. | DIED. | PR.CT.MORT. |
|---|---|---|---|
| American Cases | 19 | 6 | 32 |
| Foreign Cases | 13 | 3 | 23 |
| Totals | 32 | 9 | 28 |

Dr. Brinton compares the mortality of amputation at the knee joint with that of amputations of the thigh, in order to show that the knee joint is much the safer location. In doing so, however, he commits the mistake of making his comparison with amputations in all parts of the thigh together. This is not fair. Cases which require amputation at the middle and upper thirds of the thigh, do not admit of the knee joint as a substitute. The only thigh amputations which allow the choice are those at the lower third. If now we take our summary of amputations in the lower third abroad, and place them beside the corresponding knee amputations, we get the following result:

| | MORTALITY OF AMPUTATIONS. At Lower 3d of Thigh. | At Knee Joint. |
|---|---|---|
| Traumatic Primary | 45 per cent. | 48 per cent. |
| Traumatic Secondary | 60 " " | 49 " " |
| Pathological | 20 " " | 26 " " |
| Averages | 42 " " | 41 " " |

The superiority of the knee joint amputation over that at lower third of the thigh averages only one per cent., a difference too small to be trusted, especially as part of the cases are picked up from scattering operations reported in journals, a method of collection which always gives too large a proportion of successful results.

Mr. Liston, in Holmes' System of Surgery, p. 606, praises the operation exceedingly for the small amount of tissue divided, the excellence of the flap, the absence of exfoliation of bone and great length and usefulness of the stump.

T. Holmes (Surgery, its Principles and Practice, p. 923,) says he is "rather fond" of the operation.

Gant's Surgery, p. 706, says the "results with regard to the stump are advantageous."

Gross' System of Surgery, Vol. II., p. 1122, praises the operation because of its length of stump, the fact that it avoids some dangers by not opening the medullary canal, has no exfoliation of bone, and has a low mortality; but he opposes its performance when any point lower down is admissible.

Parker, of New York, in the *New York Journal of Medicine*, Vol. IX., N. S., p. 308, advocates it as "justifiable," and giving a good stump.

Dr. Henry Smith, in his Operative Surgery, at first opposed it, but later, in his Principles and Practice of Surgery, Vol. II., p. 704, retracted his first opinion, and though still in doubt, inclined to favor it.

Markoe of New York, Brinton of Philadelphia, Erichsen of London, and Von Langenbeck of Berlin, all favor the operation.

Of the older surgeons, Velpeau, Textor, Kern, Volpi, Brasdor, J. L. Petit, Hoin and Blandin advocate the operation; while Dupuytren, Larrey and Zang unconditionally opposed it.

### CONCLUSIONS.

It is evident that the mass of the best writers now favor the amputation at the knee joint whenever the condition admits of its being substituted for that of the lower third of the thigh. Its danger is perhaps a little less, and the stump is better. It is therefore to be preferred in such cases. As to the question whether simple disarticulation, or sawing through the condyles is the best, the statistics show the danger to be the same, when the American and Foreign cases are added together. It has been thought that the presence of the cartilage and synovial surface would have some of the same evils which result from opening a knee joint to the air, but experience apparently shows otherwise, or if the presence of those tissues is somewhat objectionable, that danger is balanced by the increased risk of pyaemia induced when the cancellous tissue of the condyles is sawn through.

### AMPUTATION OF THE LEG.

Of this very common operation I find the following cases recorded in the Lake States:

### TABLE IX.

### AMPUTATION OF THE LEG IN THE LAKE STATES.

| No. Andrew's Sur.Rec | OPERATOR. | Age. | REASON FOR OPERATION. | COMPLICATIONS. | Operati'n | Cond. at opr | Time to operation. | Result. | Time to death or recovery | Practice. |
|---|---|---|---|---|---|---|---|---|---|---|
| 644 | Dr. E. Andrews | 35 | Both feet crushed | Mortification | Dbl. Amp | Good | | Recover'd | | Private |
| 1553 | " " " | -- | | | Middle 3d | | | " | 8 mos. | " |
| 1601 | " " " | -- | Leg crushed by cars | Kept in close, foul room. | " " | Good | Primary | Died | | " |
| 1857 | " " " | 36 | Neuralgic stump of leg | None | Re-a.up. 3 | " | 4 years | Recover'd | 30 days | Hospital. |
| 1943 | " " " | 7 | Leg and arm crushed by cars | Great shock—amp.of arm | Middle 3d | Bad | Primary | Died | 2 " | " |
| 5390 | " " " | 57 | Large ulcer from burn | None | Lower 3d | Good | 2 years | Recover'd | 6 weeks | " |
| 5392 | " " " | -- | Fractured leg by cars | | Middle 3d | " | Primary | " | 10 " | " |
| 5508 | " " " | -- | Fractured leg by cars | | Circ.m.3d | " | " | " | 8 " | " |
| 5567 | " " " | -- | Foot crushed off | None | Upper 3d | " | " | " | 8 mos. | " |
| 5661 | " " " | -- | Leg crushed by cars | Severe hemorrhage | " " | Med. | " | " | 5 weeks. | " |
| 5196 | " " " | 35 | Leg crushed by cars | Mortification of foot | Middle 3d | Bad | 3 days | Died | 6 days | " |
| 6017 | " " " | 45 | Comp.fract.foot&leg. Caries of tibia | None | " " | " | Secondary | Recover'd | | Private |
| 6042 | " " " | 60 | Caries of tibia | None | Upper 3d | " | Several yrs. | " | | " |
| 6200 | " " " | 55 | Caries of ankle | Calcified arteries, exhaus | Middle 3d | " | " weeks | Died | 3 days | " |
| | " La Count | 25 | Comp. fract. of ankle | None | Cir. low.3 | Good | Primary | Recover'd | | " |
| | " " " | 45 | Foot and ankle torn badly | None | " " | " | " | " | | " |
| | " " " | 30 | Wound of ant. tibial art. Gangrene | Hemorrhage | " mid.3 | Bad | Secondary | " | | " |
| | " " " | 16 | Comp. fract. tibia and fibula | None | " low. 3 | Med. | Primary | " | | " |
| 6600 | " E. Andrews | 25 | Diseased foot from injury | None | Flap low3 | " | Secondary | " | 6 weeks | Hospital. |
| 6975 | " " " | -- | Comp. and commin. fract. of leg | | Circ. | | Primary | " | 22 " | " |
| 7012 | " " " | 62 | Senile gangrene | Weak with age | Flap up 3 | Bad | 1 month | Died | 5 days | " |
| | " " " | 30 | Both feet crushed | Great shock | " low. 3 | " | Primary | " | 1 " | Private |
| | " D. Brainard | 12 | Caries of ankle | | Lower 3d | | | Recover'd | | |
| | " " " | -- | Comp. fract. of ankle | | " " | | Primary | " | | Hospital. |
| | " Ammerman | 53 | Necrosis of tibia and fibula | | Flap up. 3 | | | " | 36 days | " |
| | " A. Fisher | 45 | Comp. and commin. fract. leg & arm | Great shock, ampt.of arm | Upper 3d | Bad | 4 hours | Died | 6 hours | Private |
| | " " " | 20 | Comp. and commin. fract. leg, R.R. | | " " | Med. | 12 " | Recover'd | | " |
| | " E. Owens | -- | R. R. fracture | | " " | | Primary | " | | Hospital. |
| | " " " | -- | Disease of parts | | " " | | | " | | " |
| | Cook Co. Hosp. | -- | | | Middle 3d | | Secondary | " | | " |
| | " " " | -- | | | Lower 3d | | Primary | " | | " |
| | " " " | -- | | | " " | | Secondary | Died | | " |
| | " H. Wardner | 17 | Leg crushed by falling tree | Shock and contusion of pelvis, thigh&abdomen | Upper 3d | Bad | Primary | " | 4 days | Private |
| | " " " | 11 | Leg crushed on R.R.and fract.elbow | Shock | Middle 3d | Med. | " | Recover'd | 2 mos. | " |
| | " " " | 25 | Foot and ankle crushed by boats | Shock and hemorrhage | Lower 3d | " | " | " | 6 weeks | " |

### Table IX.—Continued.

| | | | | | | | | | | |
|---|---|---|---|---|---|---|---|---|---|---|
| -------- | Dr. N. Senn | 10 | Ankle cut by reaper | Shock and hemorrhage | Lower 3d | ------ | 6 hours | Recover'd | 6 weeks | Private |
| -------- | " " " | 28 | Caries of ankle and tarsus fr. injury | | " " | Bad | 1 year | " | 4 " | " |
| -------- | " " " | 21 | Leg crushed by runaway | Shock and hemorrhage | Circ.m.3d | ------ | 8 hours | " | 4 " | " |
| -------- | " H. Wardner | 25 | Ankle cut by axe | | Upper 3d | Bad | 3 days | " | 4 " | " |
| -------- | " " " | 35 | Caries of ankle | Tuberculous diathesis | Middle 3d | " | 1 year | " | 6 " | " |
| -------- | " M. Waterhouse | 12 | Leg crushed by car wheels | None | Upper 3d | Good | Primary | " | 21 days | " |
| -------- | " " " | 75 | Leg crushed by wagon wheels | Anæmia | " " | Bad | " | Died | 3 " | " |
| -------- | " E. D. Kittoe | 16 | Leg torn by threshing machine | Great shock | " " | " | 8 hours | Recover'd | | " |
| -------- | " " " | 40 | Leg torn by threshing machine | None | " " | Good | 6 " | " | | " |
| -------- | " E. Kittoe, Jr. | 42 | Bad comp. and commin. fract. of leg | Intemperate | " " | Bad | 3 months | " | | " |
| -------- | " J. Andrews | 15 | Comp. fract. of leg | | | Good | Secondary | " | 15 days | " |
| -------- | " E. W. Lee | 30 | Comp. fract. of leg | None | Flap up. 3 | Bad | Primary | Died | 24 hours | " |
| -------- | " " " | 8 | Comp. fract. of leg | None | " " | Med. | " | Recover'd | 4 weeks | " |
| -------- | " " " | 22 | Comp. fract. of leg | None | " " | " | " | " | 14 " | " |
| -------- | " " " | 9 | Comp. fract. of leg | None | " " | " | " | Died | 10 days | " |
| -------- | " " " | 25 | Comp. fract. of leg | None | " " | " | " | " | 5 " | " |
| -------- | " " " | 45 | Comp. fract. of leg | Concussion of brain | " " | Bad | " | " | 48 hours | " |
| -------- | " E. Andrews | 22 | Feet crushed by cars | Great shock. Pirogoff's ampu. of opposite foot eight days after | Lower 3d | Bad | " | Recover'd | | Hospital |
| -------- | " S. Marks | 27 | Amurismal varix of anterior tibial vessels, caused by scythe when 13 | | Upper 3d | Good | 14 years | Died | 9 days | Hospital |
| -------- | " " " | 60 | Caries of the tarsus | | Low. 3 flap | Med. | About 6 yrs | Recover'd | 8 weeks | " |
| -------- | " " " | 8 | Gangrene of foot after R. R. accident, fracture of the other leg | | " " | Bad | 15 days | " | 4 " | " |
| -------- | " " " | 58 | Caries of the tibia | | Upper 3d | Good | 6 years | " | | Private |
| -------- | " " " | 19 | Disease of ends of bones, following primary amputation of leg | | Mid. 3 flap | " | 9 months | " | 8 " | Hospital |
| -------- | " " " | 9 | Both ankles crushed by locomotive | Both legs | Low. 3 flap | Med. | 2 hours | " | 4 " | Private |
| -------- | " " " | 41 | Both feet frozen. Drunk | " " | Mid. 3 flap | Bad | 8 days | " | 6 " | Hospital |
| -------- | " " " | 30 | Foot and ankle crushed by locomo | | Low. 3 flap | Good | Primary | " | 8 " | Private |
| -------- | " " " | 23 | Foot crushed by cars | | " " | " | Secondary | " | 6 " | " |
| -------- | " " " | 28 | Caries of tarsus | | " " | " | 3 years | " | 8 " | Hospital |
| -------- | " " " | 20 | Leg cut off with steamboat cable immediately above ankle | | " " | " | Primary | " | 3 mos. | " |
| -------- | " " " | 32 | Foot and low. port. of tibia crushed | | Mid. 3 flap | " | " | " | 4 weeks | Private |
| -------- | " " " | 58 | Caries of tarsus | | Low. 3 flap | Med. | 21 years | " | 6 " | Hospital |
| -------- | " " " | 55 | Caries of tarsus and lower end tibia | | Mid. 3 flap | " | 9 years | " | 6 " | Private |
| -------- | " " " | 19 | Caries of tibia | | Upper 3d | Bad | 3 years | " | 6 mos. | " |
| -------- | " " " | 43 | Caries of tarsus | | Low. 3 flap | " | 1 year | " | 3 mos. | Hospital |
| -------- | " " " | 42 | Caries of tarsus | | " " | Bad | 2½ years | Died | 7 days | Private |

RECAPITULATION.

| | CASES. | DIED. | PER CENT. MORTALITY. |
|---|---|---|---|
| Total of all locations | 70 | 16 | 23 |
| " upper 3d | 22 | 8 | 36 |
| " middle 3d | 16 | 4 | 25 |
| " lower 3d | 23 | 3 | 13 |
| Primary, all locations | 32 | 9 | 28 |
| Intermediary and secondary, all locations | 16 | 3 | 19 |
| Pathological, all locations | 13 | 3 | 23 |
| Upper 3d, primary | 13 | 6 | |
| " intermediary and secondary combined, | 4 | 1 | |
| " pathological | 5 | 1 | |
| Middle 3d, traumatic and primary | 7 | 2 | |
| " intermediary and secondary combined | 6 | 1 | |
| " pathological | 3 | 1 | |
| Lower 3d, traumatic, primary | 12 | 1 | |
| " intermediate and secondary combined | 6 | 1 | |
| " pathological | 5 | 1 | |
| Conditions imperfectly stated | 9 | 1 | |
| Hospital practice | 28 | 5 | 18 |
| Private practice | 41 | 11 | 27 |

These cases are not sufficiently numerous to settle all points, but they show that, like amputations of the thigh, those nearest the body are most dangerous. The upper 3d has a mortality of 36 per cent., the middle of 25 per cent., and the lower 3d of 13 per cent.

Hospital cases, by some accidental coincidence show better than those in private practice.

AMPUTATIONS OF THE LEG ABROAD.

Of these the literature of surgery furnishes a prodigious list, and, were they properly classified, they would settle nearly all questions capable of statistical solution. Unfortunately they are very imperfect in detail, and only a portion of them can be classified.

AMPUTATION OF THE LEG ABROAD; UPPER 3D, PRIMARY.

| AUTHORITIES. | CASES. | DIED. |
|---|---|---|
| Rept. Boston City Hosp., Dr. Cheever | 4 | 2 |
| Mass. Gen. Hosp. Rept., 1871 | 26 | 7 |
| Dr. Herrgolt, Siege of Strassburg, 1870–71 | 14 | 5 |
| Warren's Surgery, Confed. Army Rept. | 41 | 17 |
| Totals | 85 | 31 |

Mortality, 36 per cent.

AMPUTATION OF THE LEG ABROAD; UPPER 3D, INTERMEDIARY AND SECONDARY COMBINED.

| AUTHORITIES. | CASES. | DIED. |
|---|---|---|
| Rept. Boston City Hosp., Dr. Cheever | 1 | 0 |
| " Mass. Gen. Hosp, 1871 | 13 | 4 |
| Dr. Herrgolt, Siege of Strassburg, 1870–71 | 6 | 3 |
| Dr. E. Warren's Surg., p. 394, Confederate Army U. S. | 33 | 18 |
| Totals | 53 | 25 |

Mortality, 47 per cent.

AMPUTATION OF THE LEG ABROAD; UPPER 3D, PATHOLOGICAL.

| AUTHORITIES. | CASES. | DIED. |
|---|---|---|
| Rept. Bost. City Hosp., Dr. Cheever | 1 | 1 |
| " Mass. Gen. Hosp., | 42 | 7 |
| " Rostock Hosp. Rep | 4 | 2 |
| Statist. des Hôpitaux de Paris, 1861–62–63 | 15 | 9 |
| Totals | 62 | 19 |

Mortality, 31 per cent.

AMPUTATION OF THE LEG ABROAD; MIDDLE 3D, PRIMARY.

| AUTHORITIES. | CASES. | DIED. |
|---|---|---|
| Rept. Boston City Hosp., Dr. Cheever | 9 | 5 |
| " Mass. Gen. Hosp., 1871 | 47 | 19 |
| " U. S. Marine Hosps., Dr. Woodworth | 1 | 1 |
| Dr. Herrgolt, Siege of Strassburg, 1870–71 | 2 | 0 |
| Dr. E. Warren's Surg., p. 394, Confederate Army U. S. | 8 | 5 |
| Totals | 67 | 30 |

Mortality, 45 per cent.

AMPUTATION OF THE LEG ABROAD; MIDDLE 3D, INTERMEDIARY AND SECONDARY COMBINED.

| AUTHORITIES. | CASES. | DIED. |
|---|---|---|
| Rept. Boston City Hosp., Dr. Cheever | 3 | 1 |
| " Mass. Gen. Hosp., 1871 | 20 | 8 |
| Dr. Herrgolt, Siege of Strassbugh, 1870–71 | 1 | 1 |
| Dr. E. Warren's Surg., p. 394, Confed. Army | 3 | 2 |
| Totals | 27 | 12 |

Mortality, 44 per cent.

AMPUTATION MIDDLE 3D OF LEG ABROAD, PATHOLOGICAL.

| AUTHORITIES. | CASES. | DIED. |
|---|---|---|
| Rept. Boston City Hosp., Dr. Cheever | 3 | 0 |
| " Mass. Gen. " 1871 | 46 | 3 |
| Totals | 49 | 3 |

Mortality, 6 per cent.

AMPUTATION LOWER 3D OF LEG ABROAD, PRIMARY.

| AUTHORITIES. | CASES. | DIED. |
|---|---|---|
| Rept. Boston City Hosp., Dr. Cheever | 3 | 0 |
| " Mass. Gen. Hosp., 1871 | 20 | 7 |
| Dr. Herrgolt, Siege of Strassburg, 1870–71 | 2 | 1 |
| Leeds Gen. Infirmary, Dr. Nunneley | 69 | 28 |
| Dr. E. Warren's Surg. Confederate Army, p. 394 | 5 | 1 |
| Totals | 99 | 37 |

Mortality, 37 per cent.

AMPUTATION OF LOWER 3D OF LEG ABROAD, INTERMEDIARY AND SECONDARY COMBINED.

| AUTHORITIES. | CASES. | DIED. |
|---|---|---|
| Rept. Boston City Hospt. | 6 | 1 |
| " Mass. Gen. Hosp. 1871 | 15 | 5 |
| " U. S. Marine Hosp. | 2 | 0 |
| Dr. Herrgolt, Siege of Strassburg, 1870–71 | 2 | 1 |
| Dr. E. Warren's Surg., p. 394, Confederate Army | 6 | 2 |
| Totals | 31 | 9 |

Mortality, 29 per cent.

AMPUTATION OF THE LOWER 3D OF LEG ABROAD, PATHOLOGICAL.

| AUTHORITIES. | CASES. | DIED. |
|---|---|---|
| Boston City Hospt. Rep. | 1 | 0 |
| Rostock Hospt. Rept. | 2 | 0 |
| Mass. Gen. Hospt. Rept. 1871 | 28 | 5 |
| Totals | 31 | 5 |

Mortality 16 per cent.

AMPUTATION OF THE LEG ABROAD, IMPERFECTLY CLASSIFIED.

| AUTHORITIES. | CASES. | DIED. |
|---|---|---|
| Circular No. 6, Surg. Gen. U. S. A. | 2348 | 611 |
| Statistics of Crimean War | 1361 | 940 |
| " Italian War | 475 | 326 |
| " German-French War | 141 | 70 |
| British Country Hospitals | 838 | 177 |
| St. Bartholomew's Hosp., 1869 | 193 | 61 |
| K. k. allg. Krankenhaus Bericht, Wien | 241 | 71 |
| Leeds General Infirmary | 99 | 15 |
| Parisian Hospitals | 266 | 160 |
| Edinburg Infirmary, 1859 to 1868 | 86 | 38 |
| Glasgow " 1844 to 1868 | 180 | 77 |
| Guy's Hospital, 1861 to 1868 | 102 | 36 |
| London " 1862 to 1868 | 67 | 39 |
| Combined Reports from various authors | 558 | 212 |
| Totals | 6955 | 2833 |

Mortality, 40 per cent.

GENERAL SUMMARY OF AMPUTATIONS OF THE LEG.

| TOTALS. | LAKE STATES. | | | ABROAD. | | |
|---|---|---|---|---|---|---|
| | CASE | DIED | PER CT. MORT | CASE | DIED | PER CT. MORT |
| Total of all kinds | 70 | 16 | 23 | 7459 | 3004 | 40 |
| " private practice | 41 | 11 | 27 | | | |
| " hospital " | 28 | 5 | 18 | | | |
| " upper 3d | 22 | 8 | 36 | 200 | 75 | 38 |
| " middle 3d | 16 | 4 | 25 | 143 | 45 | 31 |
| " lower 3d | 23 | 3 | 13 | 161 | 51 | 32 |
| Upper 3d, primary | 13 | 6 | | 85 | 31 | 36 |
| " intermediary and secondary | 4 | 1 | | 53 | 25 | 47 |
| " pathological | 5 | 1 | | 62 | 19 | 31 |
| Middle 3d, primary | 7 | 2 | | 67 | 30 | 45 |
| " intermediary and secondary | 6 | 1 | | 27 | 12 | 44 |
| " pathological | 3 | 1 | | 49 | 3 | 6 |
| Lower 3d, primary | 12 | 1 | | 99 | 37 | 37 |
| " intermediary and secondary | 6 | 1 | | 31 | 9 | 29 |
| " pathological | 5 | 1 | | 31 | 5 | 16 |

The small proportion of perfectly classified statistics renders this summary a little irregular, but still it shows the general decrease of danger as we recede from the body, and the superior safety of pathological amputations (those of "expediency" always excepted) over traumatic cases.

OPINIONS OF AUTHORS AND CONCLUSIONS.

Authors say very little about the special indications demanding amputation of the leg. In general terms they are such as demand amputation in any other part of the body. The surgeon must not be swayed, as is too often the case, by the ghastly appearance of a bad compound fracture, but consider the intrinsic condition of the limb as to circulation and innervation. If these functions are fairly preserved, a great amount of bony injury can be successfully overcome. In war a bullet traversing from before backward may shatter the tibia, bury a hundred of its fragments in the tissues of the calf, and destroy the posterior tibial artery and nerve, and yet the wound make no great external display. On the other hand, if the ball traverse from behind forward, the artery and nerve may escape, and the fragments of bone be driven out into the external air in front. The wound is large, ragged, and terrible to the eye, but much less dangerous than the former. Yet many a surgeon, in looking over his patients, has been moved by mere external appearances to amputate the better limb and try to save the worse one. Analogous errors are common in civil practice.

A mere bad compound fracture does not necessarily require amputation. Compound dislocations of the ankle often require resection, but rarely amputation. It is much disputed whether it is best to amputate anywhere for senile gangrene in the foot. If, however, it is decided to be best, the amputation should be at least as high as the upper third of the leg, and not in the foot. Some advise the lower third of the thigh.

Where the injury or disease requiring amputation of the leg admits of a choice of location, all authors agree that it should be as low down as possible, in order to reduce the risk to the lowest attainable figure. In the leg, as well as elsewhere, amputations of "expediency," *i. e.*, for deformities, etc., have the general risk of traumatic, and not the slighter one of pathological cases.

In compound fractures and dislocations of the ankle joint, amputation should not be performed unless mortification of the foot or other imperative reasons demand it. Resection has the best results in those cases, conservative treatment next, and amputation the worst.

### SYME'S AMPUTATION AT THE ANKLE.

I have not obtained a single recorded case of this operation in the Lake States, but the literature of the profession gives us many cases from abroad, though generally not classified. They are as follows:

| AUTHORITIES. | CASES. | DIED. |
|---|---|---|
| Deutsch Zeitschrift, fur Chir. Bd. I. und II. | 3 | 0 |
| Dr. Herrgolt, Seige of Strassburg, 1870–71 | 2 | 1 |
| Dr. E. Warren's Surg., Confederate Army | 2 | 1 |
| Hancock | 219 | 17 |
| Birmingham Hospt. 1858–64, Richardson | 45 | 7 |
| Rostock Hospt. Rept. 1868 | 6 | 1 |
| Brit. Army Med. Rep. | 2 | 0 |
| Archiv. Klin. Chir. Bd. X, Billroth | 2 | 0 |
| " " " " X, XIII and XVII | 44 | 3 |
| Totals | 325 | 30 |

Mortality, 9 per cent.

We will discuss the merits and opinions on this operation at the same time with the next one.

### PIROGOFF'S AMPUTATION.

Of this we find in the Lake States quite a number of cases which are given below:

### TABLE X.

### PIROGOFF'S AMPUPATION IN THE LAKE STATES.

| No. | OPERATOR OR REPORTER. | Age. | CAUSE OF OPERATION. | COMPLICATIONS. | Operation. | Con-diti'n | Time of operation | Result. | Time to death or recovery. | Practice. |
|---|---|---|---|---|---|---|---|---|---|---|
| 1866 | Dr. E. Andrews | 22 | Both feet frozen | Both feet amp. at once | Pirogoff's | Good | Second'y. | Died | 16 days | Hosp |
| 2063 | " " | .. | Foot crushed | Intemperate | " | Med | Primary | " | 51 days | " |
| 5428 | " " | .. | " " | " | " | Good | " | Recov'r'd | ------ | " |
| 5684 | " " | 14 | " " | None | " | " | " | " | 5 months | " |
| 5259 | " " | .. | " " and mortified | " | " | " | 13 days | " | 4½ ms | " |
| 1967 | H.A.Johnson | .. | " " | ------ | " | " | Primary | " | 2½ ms | " |
| 8430 | E. Andrews | 18 | Caries of tarsus | None | " | " | 18 ms | " | 12 ms | " |
| 8518 | " " | 39 | Foot crushed | " | " | Med | Primary | " | ------ | " |
| ---- | J.S.Sherman | 25 | " " | Op. leg cr. and amp. 10 d. | " | " | 10 days | " | ------ | " |

RECAPITULATION.

| | CASES. | DIED. |
|---|---|---|
| Total (all Hospital patients) | 9 | 2 |
| Primary | 5 | 1 |
| Secondary and intermediary | 3 | 1 |
| Pathological | 1 | 0 |

Total mortality in the Lake States, 22 per cent.

The cases are not numerous enough to furnish by themselves any special conclusions.

Abroad the literature furnishes us a moderate number, mostly unclassified.

PIROGOFF'S AMPUTATION ABROAD.

| AUTHORITIES. | CASES. | DIED. |
|---|---|---|
| Penn. Hosp. | 2 | 0 |
| St. George's Hosp. | 2 | 1 |
| Rostock Hospt. Rept. | 4 | 1 |
| Dr. E. Warren's surg., p. 394, Confederate Army | 1 | 0 |
| Bericht k. k. allg. Krankenhaus, Wien | 26 | 9 |
| Braithwaite's Retrospect, Jan. 1867 | 58 | 5 |
| Archiv. klin. Chir. Bd. X., Billroth | 11 | 4 |
| " " " " VIII | 1 | 0 |
| Deutsch. Zeit. f. Chir. B. I, S. 187; B. II., S. 380 | 7 | 0 |
| Totals | 112 | 20 |

Mortality, 18 per cent.

OPINIONS OF AUTHORS.

Syme's and Pirogoff's amputations are rivals of each other, being applied to the same class of cases.

The American Surgeon General's Circular, No. 6, says that Pirogoff's operation is regarded with little favor.

Baron von Horrowitz, the Surgeon-in-Chief of the Russian Marine, says that Pirogoff himself has abandoned it on account of the frequent occurrence of necrosis of the os calcis.

Dr. Stephen Smith says the stump of Syme's operation is better than that of Pirogoff.

Hewson, on the contrary, (quoted in Ashurst's Surgery, p. 122,) says that Pirogoff's stump has some decided advantages over Syme's, in that the patient can walk and run upon it.

Liston, in Holmes' System of Surgery, Vol. V., p. 644, prefers Syme's amputation as simpler, easier, and less liable to caries.

Gant's Surgery, p. 701, says this liability to caries is not present in traumatic cases.

Erichsen's Surgery, Vol. I., pp. 78, 79, speaks favorably of Pirogoff's operation, and thinks the objections to it not very well grounded in experience.

Gross' Surgery, Vol. II, p. 1119, prefers amputation of the lower 3d of the leg to either Pirogoff's or Syme's operation.

Dr. Stephen Smith, on the contrary, in his contribution to the papers of the United States Sanitary Commission, concludes that Syme's amputation is 50 per cent. safer than that at the lower 3d of the leg.

Hamilton's Surgery, p. 368, says that the stump in both Pirogoff's and Syme's operation is often most excellent.

Bryant's Surgery, p. 964, speaks in the highest terms both of Pirogoff's and Syme's amputations.

### CONCLUSIONS

As usual, the opinions of surgeons are a little contradictory of each other, yet the majority favor Syme's rather than Pirogoff's method. The statistics, though less perfect than could be desired, still point to the same conclusion, for we have as follows:

| | CASES. | DIED. | MORTALITY. |
|---|---|---|---|
| Syme's amputation | 325 | 30 | 9 per ct. |
| Pirogoff's " | 112 | 20 | 18 " |

It would appear, therefore, that thus far Pirogoff's operation has had double the mortality of Syme's, an important fact scarcely referred to by our best authors.

It is evident, therefore, that as facts and opinions now appear, they compel us to consider Syme's operation as much the best.

The military statistics of Demme, Stromeyer and Legouest, give for compound fractures of the foot much better results for conservative treatment than for any kind of amputation. The superiority of their conservative figures varies from 28 to 59 per cent.

It would seem, therefore, that an amputation of the foot is not demanded for ordinary bad compound fractures, but only for that portion of them where there is such an amount of mortification as compels it.

(To be continued.)

